# Supplementary material for: Optimizing Nitrogen Supplementation: Timing Strategies to Mitigate Waterlogging Stress in Winter- and Spring-Type Canola
Source: Plants (Basel). 2025 Aug 25;14(17):2641. doi: 10.3390/plants14172641 (PMC12430680; doi:10.3390/plants14172641)
Supplement: Supplementary file 1 [file plants-14-02641-s001.zip › plants-3667092-supplementary.pptx]

## Slide 1
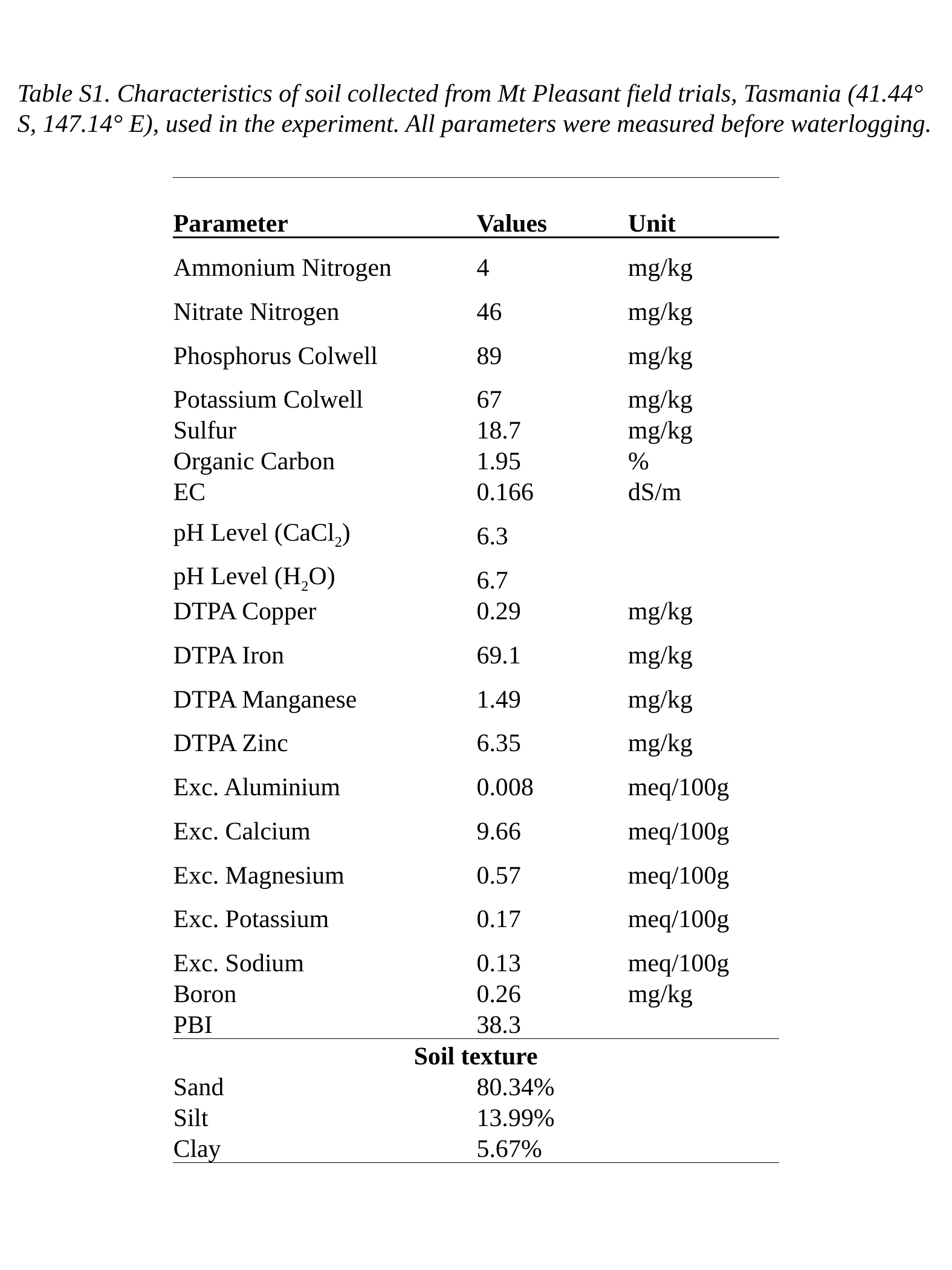

Table S1. Characteristics of soil collected from Mt Pleasant field trials, Tasmania (41.44° S, 147.14° E), used in the experiment. All parameters were measured before waterlogging.
| Parameter | | Values | Unit |
| --- | --- | --- | --- |
| Ammonium Nitrogen | | 4 | mg/kg |
| Nitrate Nitrogen | | 46 | mg/kg |
| Phosphorus Colwell | | 89 | mg/kg |
| Potassium Colwell | | 67 | mg/kg |
| Sulfur | | 18.7 | mg/kg |
| Organic Carbon | | 1.95 | % |
| EC | | 0.166 | dS/m |
| pH Level (CaCl2) | | 6.3 | |
| pH Level (H2O) | | 6.7 | |
| DTPA Copper | | 0.29 | mg/kg |
| DTPA Iron | | 69.1 | mg/kg |
| DTPA Manganese | | 1.49 | mg/kg |
| DTPA Zinc | | 6.35 | mg/kg |
| Exc. Aluminium | | 0.008 | meq/100g |
| Exc. Calcium | | 9.66 | meq/100g |
| Exc. Magnesium | | 0.57 | meq/100g |
| Exc. Potassium | | 0.17 | meq/100g |
| Exc. Sodium | | 0.13 | meq/100g |
| Boron | | 0.26 | mg/kg |
| PBI | | 38.3 | |
| Soil texture | | | |
| Sand | | 80.34% | |
| Silt | | 13.99% | |
| Clay | | 5.67% | |

## Slide 2
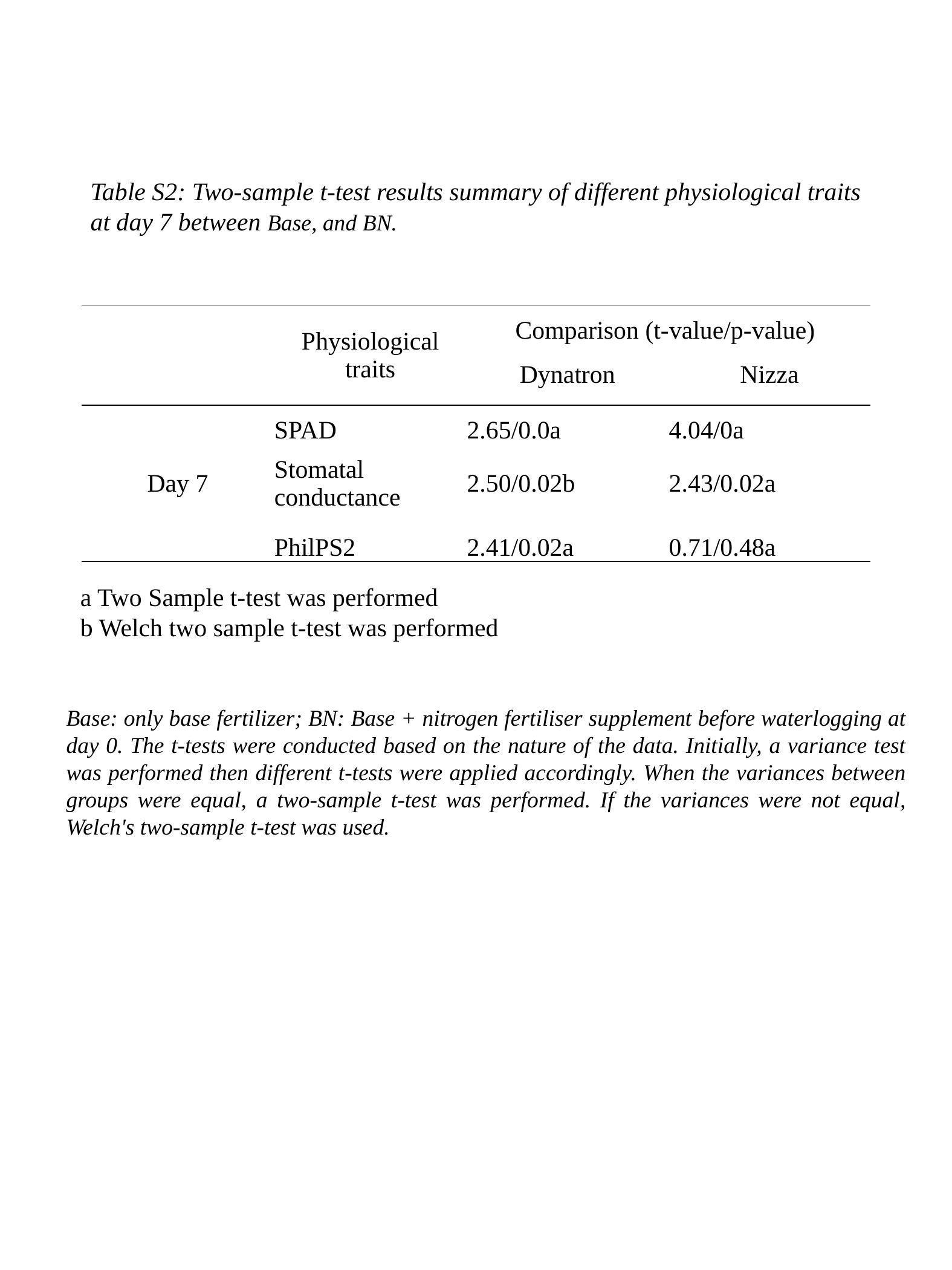

Table S2: Two-sample t-test results summary of different physiological traits at day 7 between Base, and BN.
| | Physiological traits | Comparison (t-value/p-value) | |
| --- | --- | --- | --- |
| | | Dynatron | Nizza |
| Day 7 | SPAD | 2.65/0.0a | 4.04/0a |
| | Stomatal conductance | 2.50/0.02b | 2.43/0.02a |
| | PhilPS2 | 2.41/0.02a | 0.71/0.48a |
a Two Sample t-test was performed
b Welch two sample t-test was performed
Base: only base fertilizer; BN: Base + nitrogen fertiliser supplement before waterlogging at day 0. The t-tests were conducted based on the nature of the data. Initially, a variance test was performed then different t-tests were applied accordingly. When the variances between groups were equal, a two-sample t-test was performed. If the variances were not equal, Welch's two-sample t-test was used.

## Slide 3
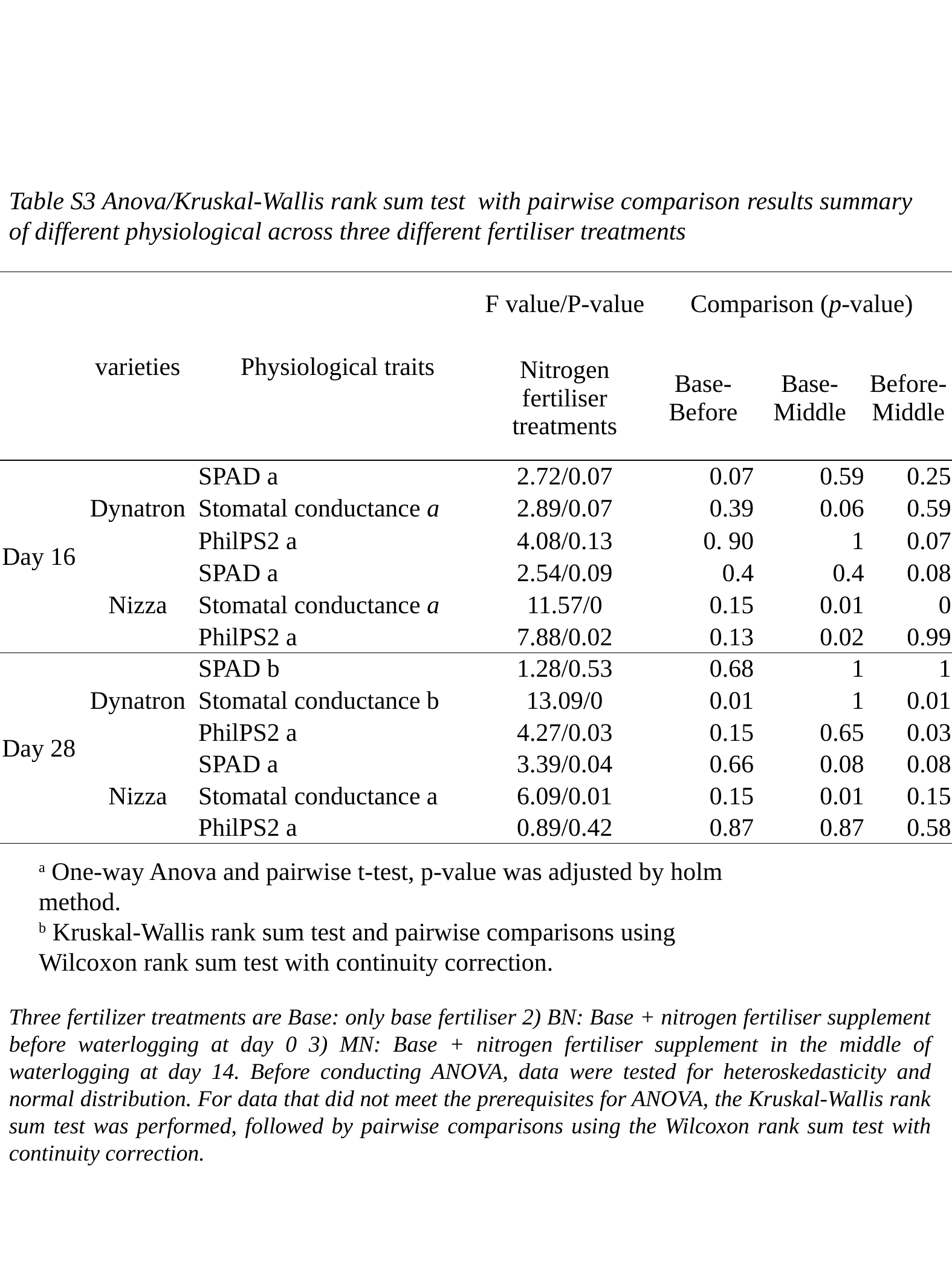

Table S3 Anova/Kruskal-Wallis rank sum test with pairwise comparison results summary of different physiological across three different fertiliser treatments
| | varieties | Physiological traits | F value/P-value | Comparison (p-value) | | |
| --- | --- | --- | --- | --- | --- | --- |
| | | | Nitrogen fertiliser treatments | Base-Before | Base-Middle | Before-Middle |
| Day 16 | Dynatron | SPAD a | 2.72/0.07 | 0.07 | 0.59 | 0.25 |
| | | Stomatal conductance a | 2.89/0.07 | 0.39 | 0.06 | 0.59 |
| | | PhilPS2 a | 4.08/0.13 | 0. 90 | 1 | 0.07 |
| | Nizza | SPAD a | 2.54/0.09 | 0.4 | 0.4 | 0.08 |
| | | Stomatal conductance a | 11.57/0 | 0.15 | 0.01 | 0 |
| | | PhilPS2 a | 7.88/0.02 | 0.13 | 0.02 | 0.99 |
| Day 28 | Dynatron | SPAD b | 1.28/0.53 | 0.68 | 1 | 1 |
| | | Stomatal conductance b | 13.09/0 | 0.01 | 1 | 0.01 |
| | | PhilPS2 a | 4.27/0.03 | 0.15 | 0.65 | 0.03 |
| | Nizza | SPAD a | 3.39/0.04 | 0.66 | 0.08 | 0.08 |
| | | Stomatal conductance a | 6.09/0.01 | 0.15 | 0.01 | 0.15 |
| | | PhilPS2 a | 0.89/0.42 | 0.87 | 0.87 | 0.58 |
a One-way Anova and pairwise t-test, p-value was adjusted by holm method.
b Kruskal-Wallis rank sum test and pairwise comparisons using Wilcoxon rank sum test with continuity correction.
Three fertilizer treatments are Base: only base fertiliser 2) BN: Base + nitrogen fertiliser supplement before waterlogging at day 0 3) MN: Base + nitrogen fertiliser supplement in the middle of waterlogging at day 14. Before conducting ANOVA, data were tested for heteroskedasticity and normal distribution. For data that did not meet the prerequisites for ANOVA, the Kruskal-Wallis rank sum test was performed, followed by pairwise comparisons using the Wilcoxon rank sum test with continuity correction.

## Slide 4
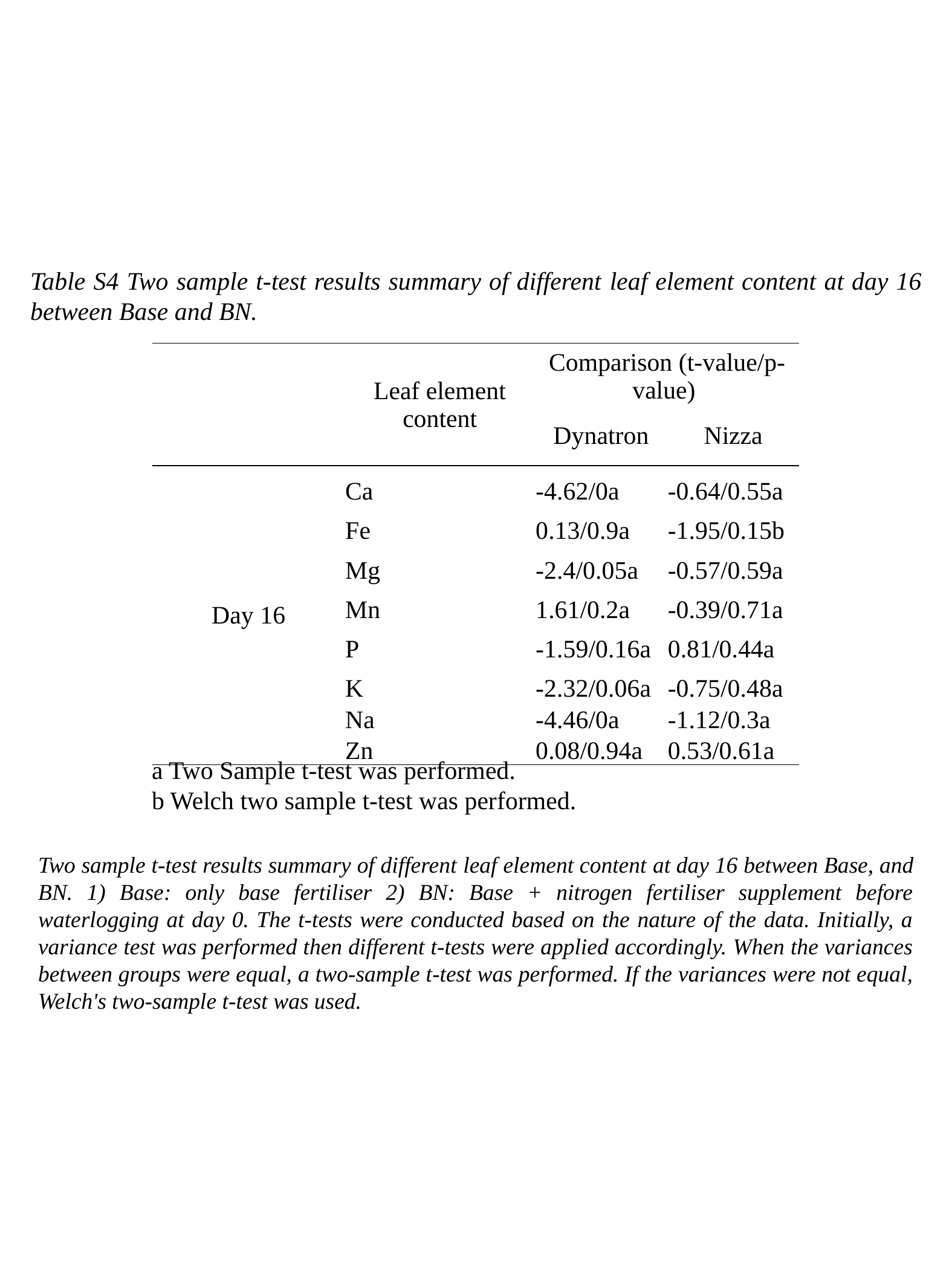

Table S4 Two sample t-test results summary of different leaf element content at day 16 between Base and BN.
| | Leaf element content | Comparison (t-value/p-value) | |
| --- | --- | --- | --- |
| | | Dynatron | Nizza |
| Day 16 | Ca | -4.62/0a | -0.64/0.55a |
| | Fe | 0.13/0.9a | -1.95/0.15b |
| | Mg | -2.4/0.05a | -0.57/0.59a |
| | Mn | 1.61/0.2a | -0.39/0.71a |
| | P | -1.59/0.16a | 0.81/0.44a |
| | K | -2.32/0.06a | -0.75/0.48a |
| | Na | -4.46/0a | -1.12/0.3a |
| | Zn | 0.08/0.94a | 0.53/0.61a |
a Two Sample t-test was performed.
b Welch two sample t-test was performed.
Two sample t-test results summary of different leaf element content at day 16 between Base, and BN. 1) Base: only base fertiliser 2) BN: Base + nitrogen fertiliser supplement before waterlogging at day 0. The t-tests were conducted based on the nature of the data. Initially, a variance test was performed then different t-tests were applied accordingly. When the variances between groups were equal, a two-sample t-test was performed. If the variances were not equal, Welch's two-sample t-test was used.

## Slide 5
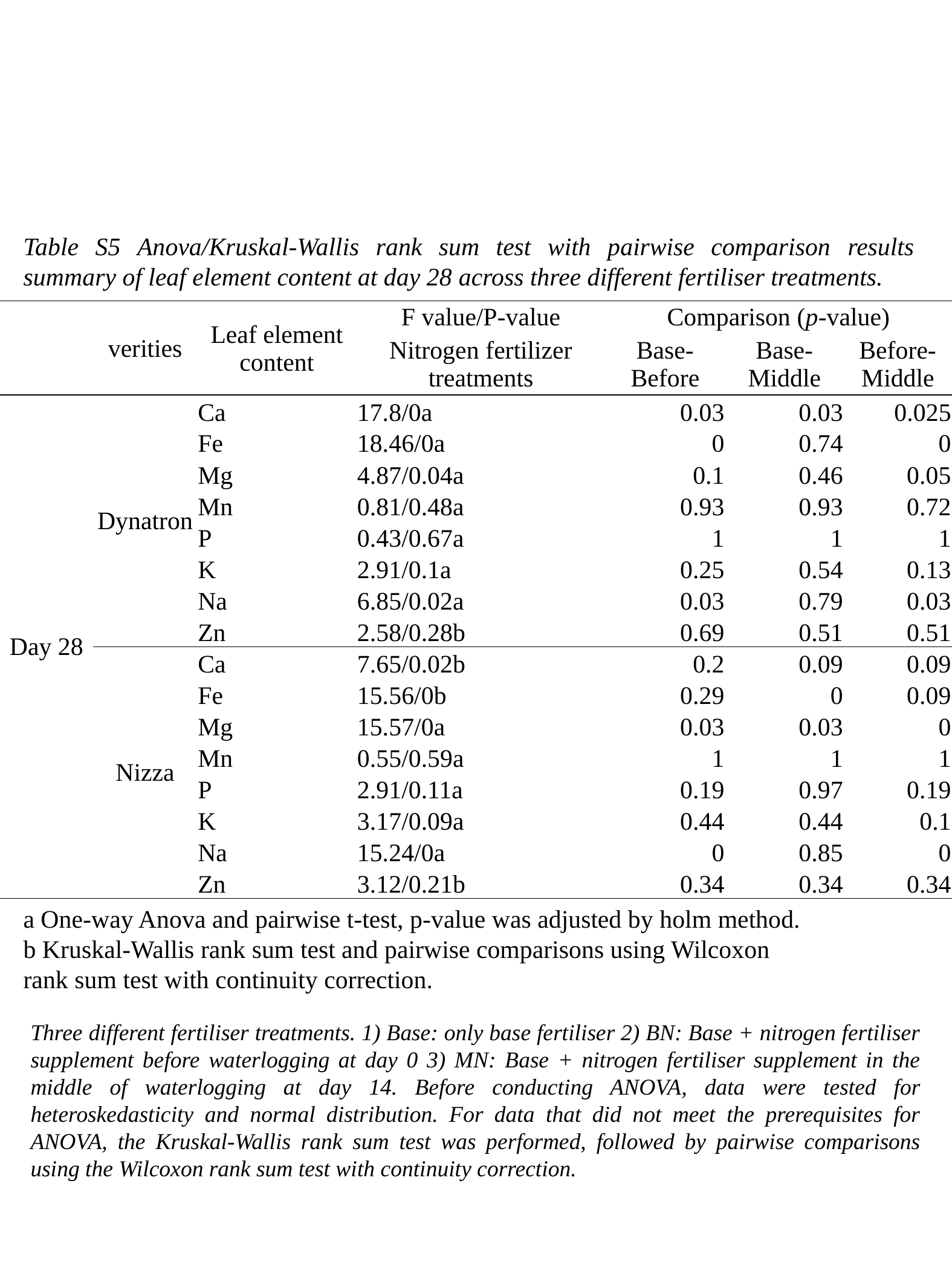

Table S5 Anova/Kruskal-Wallis rank sum test with pairwise comparison results summary of leaf element content at day 28 across three different fertiliser treatments.
| | verities | Leaf element content | F value/P-value | Comparison (p-value) | | |
| --- | --- | --- | --- | --- | --- | --- |
| | | | Nitrogen fertilizer treatments | Base-Before | Base-Middle | Before-Middle |
| Day 28 | Dynatron | Ca | 17.8/0a | 0.03 | 0.03 | 0.025 |
| | | Fe | 18.46/0a | 0 | 0.74 | 0 |
| | | Mg | 4.87/0.04a | 0.1 | 0.46 | 0.05 |
| | | Mn | 0.81/0.48a | 0.93 | 0.93 | 0.72 |
| | | P | 0.43/0.67a | 1 | 1 | 1 |
| | | K | 2.91/0.1a | 0.25 | 0.54 | 0.13 |
| | | Na | 6.85/0.02a | 0.03 | 0.79 | 0.03 |
| | | Zn | 2.58/0.28b | 0.69 | 0.51 | 0.51 |
| | Nizza | Ca | 7.65/0.02b | 0.2 | 0.09 | 0.09 |
| | | Fe | 15.56/0b | 0.29 | 0 | 0.09 |
| | | Mg | 15.57/0a | 0.03 | 0.03 | 0 |
| | | Mn | 0.55/0.59a | 1 | 1 | 1 |
| | | P | 2.91/0.11a | 0.19 | 0.97 | 0.19 |
| | | K | 3.17/0.09a | 0.44 | 0.44 | 0.1 |
| | | Na | 15.24/0a | 0 | 0.85 | 0 |
| | | Zn | 3.12/0.21b | 0.34 | 0.34 | 0.34 |
a One-way Anova and pairwise t-test, p-value was adjusted by holm method.
b Kruskal-Wallis rank sum test and pairwise comparisons using Wilcoxon rank sum test with continuity correction.
Three different fertiliser treatments. 1) Base: only base fertiliser 2) BN: Base + nitrogen fertiliser supplement before waterlogging at day 0 3) MN: Base + nitrogen fertiliser supplement in the middle of waterlogging at day 14. Before conducting ANOVA, data were tested for heteroskedasticity and normal distribution. For data that did not meet the prerequisites for ANOVA, the Kruskal-Wallis rank sum test was performed, followed by pairwise comparisons using the Wilcoxon rank sum test with continuity correction.

## Slide 6
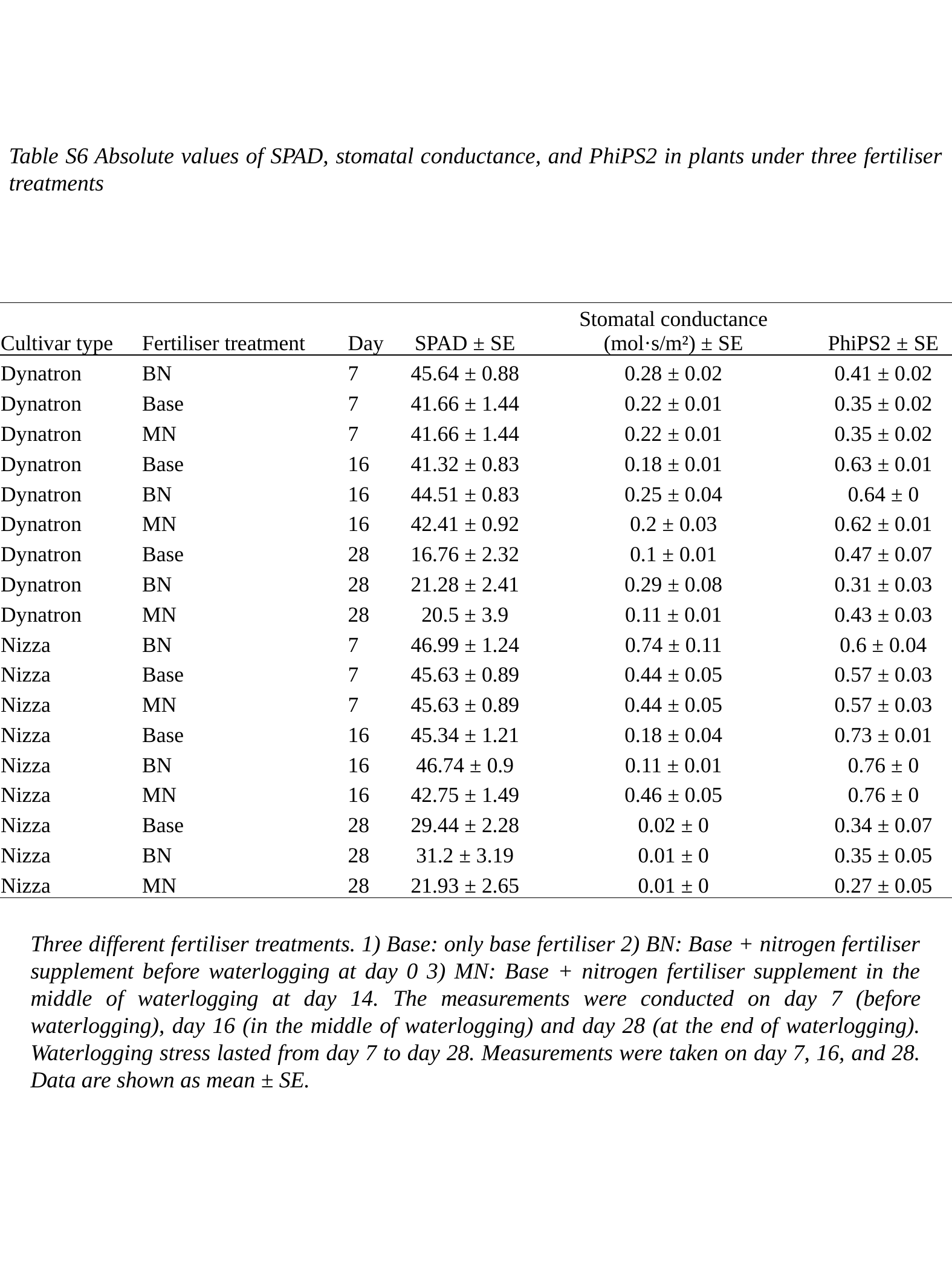

Table S6 Absolute values of SPAD, stomatal conductance, and PhiPS2 in plants under three fertiliser treatments
| Cultivar type | Fertiliser treatment | Day | SPAD ± SE | Stomatal conductance (mol·s/m²) ± SE | PhiPS2 ± SE |
| --- | --- | --- | --- | --- | --- |
| Dynatron | BN | 7 | 45.64 ± 0.88 | 0.28 ± 0.02 | 0.41 ± 0.02 |
| Dynatron | Base | 7 | 41.66 ± 1.44 | 0.22 ± 0.01 | 0.35 ± 0.02 |
| Dynatron | MN | 7 | 41.66 ± 1.44 | 0.22 ± 0.01 | 0.35 ± 0.02 |
| Dynatron | Base | 16 | 41.32 ± 0.83 | 0.18 ± 0.01 | 0.63 ± 0.01 |
| Dynatron | BN | 16 | 44.51 ± 0.83 | 0.25 ± 0.04 | 0.64 ± 0 |
| Dynatron | MN | 16 | 42.41 ± 0.92 | 0.2 ± 0.03 | 0.62 ± 0.01 |
| Dynatron | Base | 28 | 16.76 ± 2.32 | 0.1 ± 0.01 | 0.47 ± 0.07 |
| Dynatron | BN | 28 | 21.28 ± 2.41 | 0.29 ± 0.08 | 0.31 ± 0.03 |
| Dynatron | MN | 28 | 20.5 ± 3.9 | 0.11 ± 0.01 | 0.43 ± 0.03 |
| Nizza | BN | 7 | 46.99 ± 1.24 | 0.74 ± 0.11 | 0.6 ± 0.04 |
| Nizza | Base | 7 | 45.63 ± 0.89 | 0.44 ± 0.05 | 0.57 ± 0.03 |
| Nizza | MN | 7 | 45.63 ± 0.89 | 0.44 ± 0.05 | 0.57 ± 0.03 |
| Nizza | Base | 16 | 45.34 ± 1.21 | 0.18 ± 0.04 | 0.73 ± 0.01 |
| Nizza | BN | 16 | 46.74 ± 0.9 | 0.11 ± 0.01 | 0.76 ± 0 |
| Nizza | MN | 16 | 42.75 ± 1.49 | 0.46 ± 0.05 | 0.76 ± 0 |
| Nizza | Base | 28 | 29.44 ± 2.28 | 0.02 ± 0 | 0.34 ± 0.07 |
| Nizza | BN | 28 | 31.2 ± 3.19 | 0.01 ± 0 | 0.35 ± 0.05 |
| Nizza | MN | 28 | 21.93 ± 2.65 | 0.01 ± 0 | 0.27 ± 0.05 |
Three different fertiliser treatments. 1) Base: only base fertiliser 2) BN: Base + nitrogen fertiliser supplement before waterlogging at day 0 3) MN: Base + nitrogen fertiliser supplement in the middle of waterlogging at day 14. The measurements were conducted on day 7 (before waterlogging), day 16 (in the middle of waterlogging) and day 28 (at the end of waterlogging). Waterlogging stress lasted from day 7 to day 28. Measurements were taken on day 7, 16, and 28. Data are shown as mean ± SE.

## Slide 7
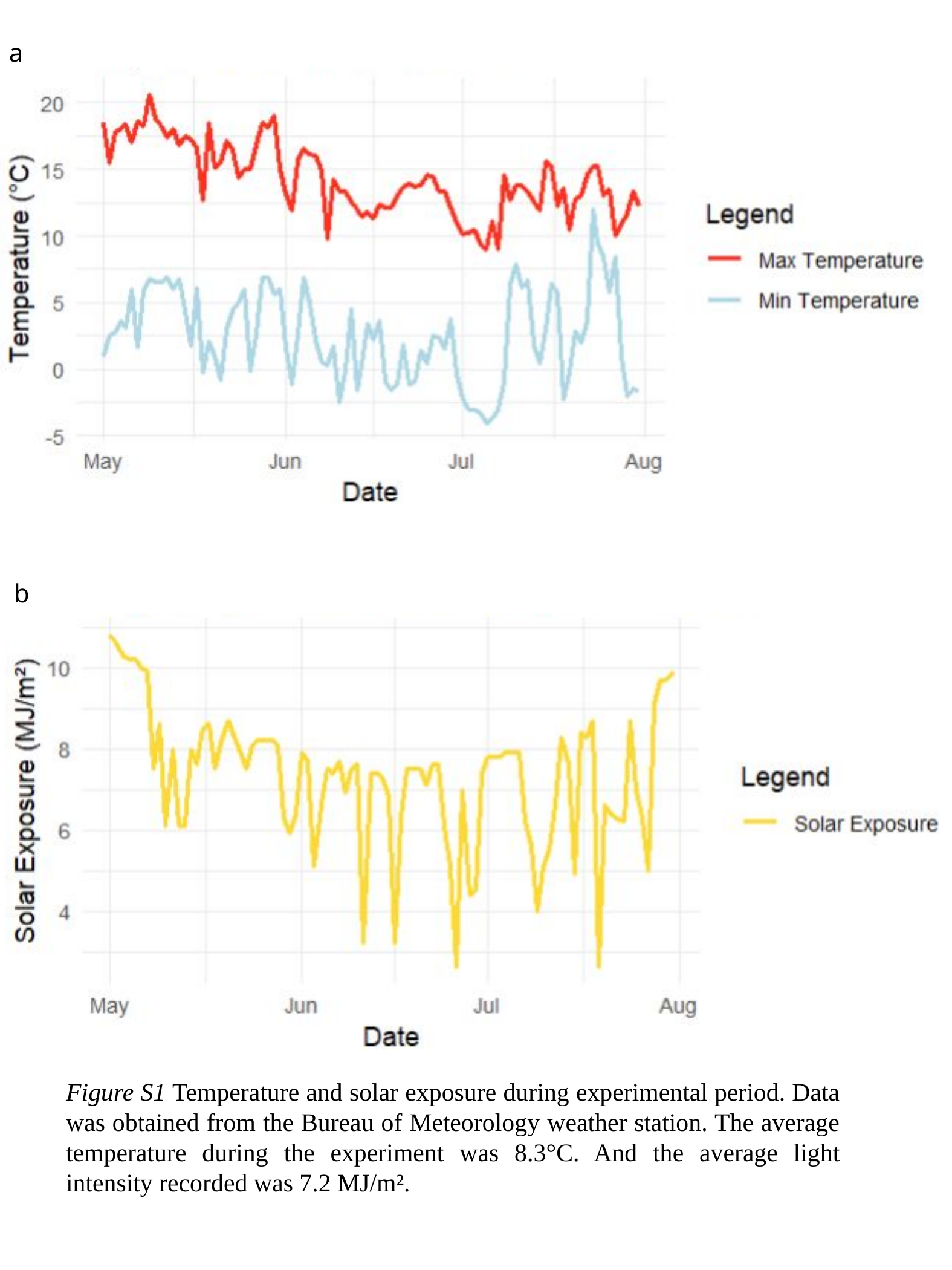

a
b
Figure S1 Temperature and solar exposure during experimental period. Data was obtained from the Bureau of Meteorology weather station. The average temperature during the experiment was 8.3°C. And the average light intensity recorded was 7.2 MJ/m².

## Slide 8
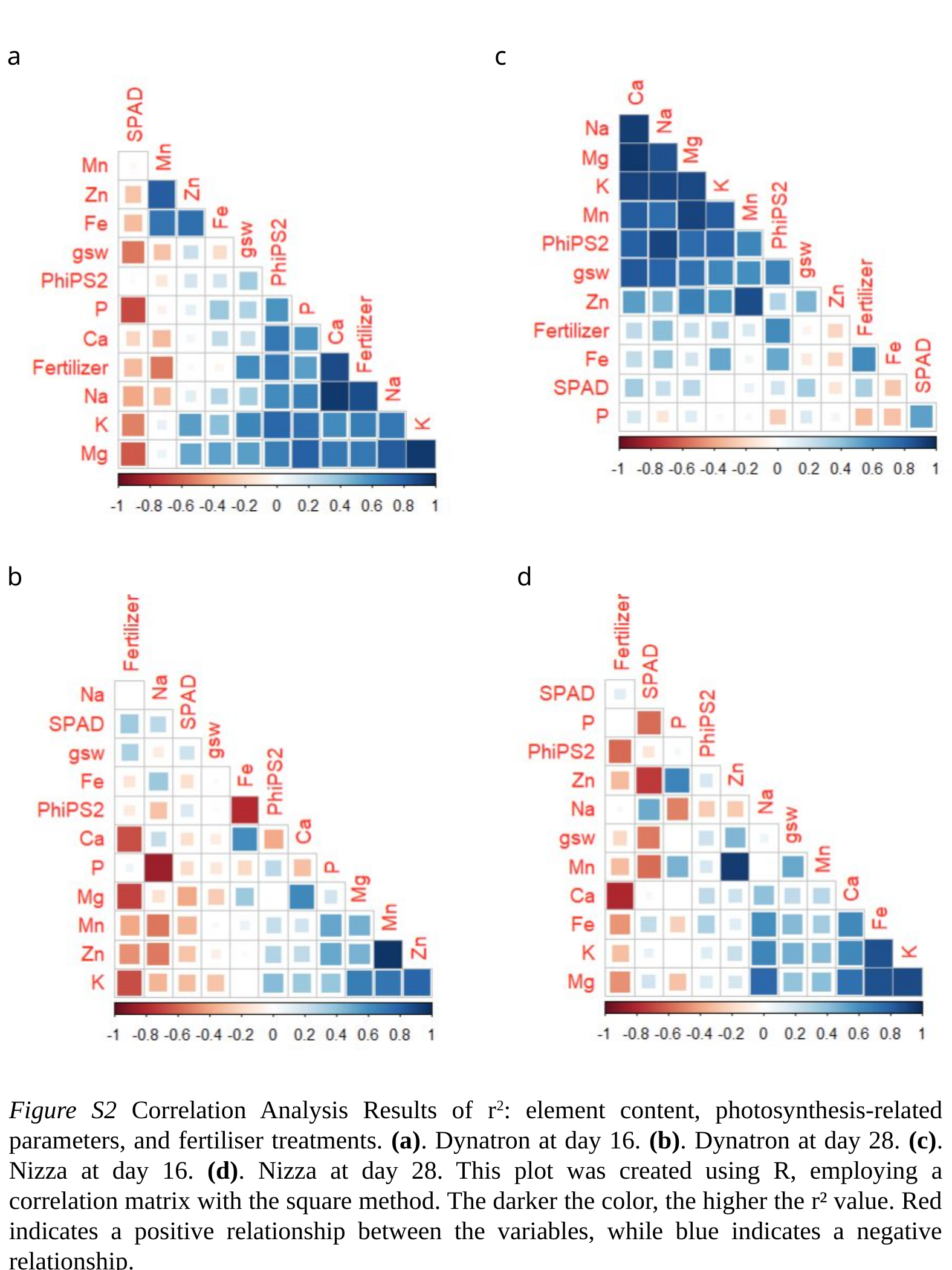

a
c
b
d
Figure S2 Correlation Analysis Results of r2: element content, photosynthesis-related parameters, and fertiliser treatments. (a). Dynatron at day 16. (b). Dynatron at day 28. (c). Nizza at day 16. (d). Nizza at day 28. This plot was created using R, employing a correlation matrix with the square method. The darker the color, the higher the r² value. Red indicates a positive relationship between the variables, while blue indicates a negative relationship.

## Slide 9
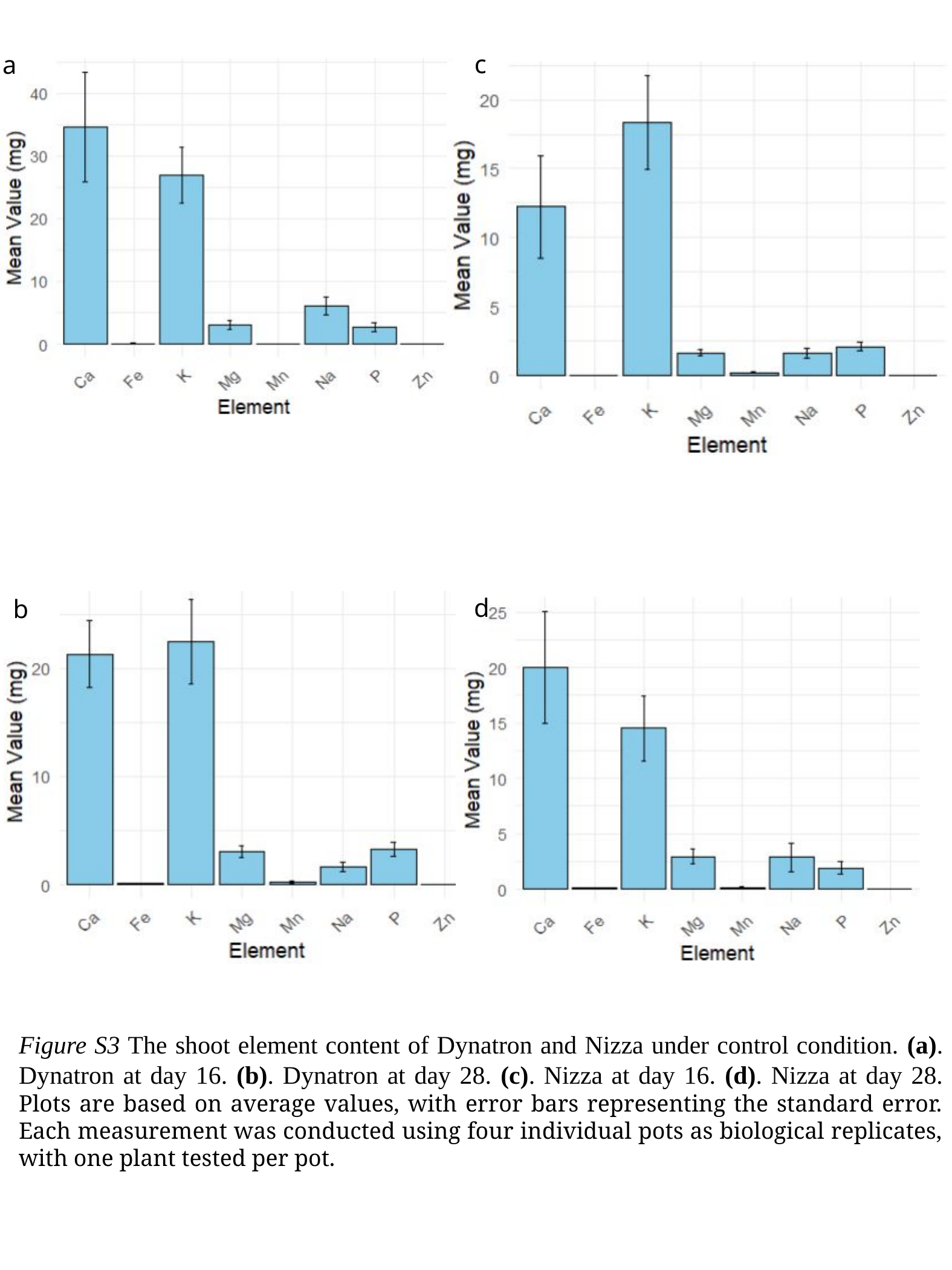

c
a
d
b
Figure S3 The shoot element content of Dynatron and Nizza under control condition. (a). Dynatron at day 16. (b). Dynatron at day 28. (c). Nizza at day 16. (d). Nizza at day 28. Plots are based on average values, with error bars representing the standard error. Each measurement was conducted using four individual pots as biological replicates, with one plant tested per pot.
